# Aquaporins and The Nervous System: from Bench to Bedside

**DOI:** 10.2174/157015910791233169

**Published:** 2010-06

**Authors:** Rita Rezzani, Luigi F. Rodella

Water is the single most abundant substance in cells and organisms and is an important molecule involved in several biochemical processes present in living cells. In humans 60-70% of body weight is water which equilibrates across the lipid bilayer in cell membranes. Forty years ago, a small number of scientists argued that specialized water-selective pores are necessary to explain the high water permeability of red blood cells and renal tubules. Therefore, the molecular identification of a 28 kDa integral membrane protein in these cells has characterized a new stream of research. Aquaporins (AQPs) are membrane proteins that transport water and, in some cases, also small solutes such as glycerol and urea. Each subtype has its own cellular distribution and distinct regulatory mechanisms of their expression. Their classical role in facilitating trans-epithelial fluid transport is well understood, as in the urinary concentrating mechanism and gland fluid secretion, while the molecular mechanisms to regulate water permeability in the nervous system are still unclear. Maintenance of the ionic and osmotic composition and volume of interstitial, glial and neuronal compartments within the nervous system is essential for normal function. Small changes in intracellular or extracellular ion or solute composition can dramatically modify bi-directional water pathway between the brain and blood vessels and alter cerebrospinal fluid formation, neural signal transduction and information processing. To date, only some AQP isoforms (AQP1, 3, 4, 5, 8, 9) have been reported in the central nervous system being identified in choroidal cells (AQP1), astrocytes (AQP1, 3, 4, 5, 8, 9), oligodendrocytes (AQP8), neurons (AQP1, 5, 8), tanycytes (AQP9) and ependymal cells (AQP1, 4, 9). In contrast to numerous studies of AQP localization and function in the central nervous system, little information is available on the expression and function of AQPs in peripheral nervous system. This issue includes six review articles in which the authors report and explore the recent findings about the involvement of AQPs both in peripheral and central nervous system.

The paper by B. Buffoli summarizes the data about the structure, regulation and function of AQPs, giving more importance to their involvement in the nervous system and underlying the development of new methods for diagnosis and therapy diseases.

The review of R. Albertini and R. Bianchi is focused on the different isoforms of AQP protein that have been identified in glial cells in central and peripheral nervous system and in reactive microglial. The chapter supports the idea that AQPs are involved in water homeostasis during different glial cell functions, such as differentiation, metabolism and excitability of neurons.

F. Bonomini and R. Rezzani emphasize the role of some AQPs present in glial cells in the maintenance or/and in the regulatory mechanisms of blood brain barrier.

On the basis of the role of AQPs in brain edema, a personal account of the role of AQPs is then presented by C. Loreto and E. Reggio, who summarized the implication of different isoforms of these proteins in relation with vascular diseases and nervous system.

In the literature, there is a lot of evidence that indicates a correlation between the expression of AQPs and the development of neurodegenerative diseases in which preservation of brain homeostasis is at risk. The review of E. Foglio and L.F. Rodella was to consider this topic concentrating on some neurodegenerative diseases, such as Neuromyielitis Optica, Alzheimer’s Diseases, Parkinson’s Diseases, Amyotrophic lateral sclerosis, Transmissible Spongiform Encephalopathies.

Recent evidence suggests a novel role of AQPs in pain transmission both in the central and peripheral nervous system. In this issue, E. Borsani reports the modulation of AQPs both in inflammatory and neuropathic pain considering different animal models and knock-out animals.

In the future, the numerous ongoing studies will certainly reveal other multifunctional roles of these proteins in humans. These roles might be exploited clinically by the development of drugs to alter AQP expression or function that could serve in the treatment of different diseases associated to peripheral and central nervous system.

